# Six rapid assessments of alcohol and other substance use in populations displaced by conflict

**DOI:** 10.1186/1752-1505-5-1

**Published:** 2011-02-11

**Authors:** Nadine Ezard, Edna Oppenheimer, Ann Burton, Marian Schilperoord, David Macdonald, Moruf Adelekan, Abandokoth Sakarati, Mark van Ommeren

**Affiliations:** 1 Faculty of Public Health and Policy, London School of Hygiene and Tropical Medicine, London, UK; 2 Independent consultant, Bangkok, Thailand; 3 United Nations High Commissioner for Refugees, Dadaab, Kenya; 4 Division of Programme Support and Management, Public Health and HIV Section, United Nations High Commissioner for Refugees, Geneva, Switzerland; 5 International Drugs and Development Advisor, Perthshire, UK; 6 Consultant Psychiatrist, Royal Blackburn Hospital, Blackburn, UK; 7 United Nations High Commissioner for Refugees, Jakarta, Indonesia; 8 Department of Mental Health and Substance Abuse, World Health Organization, Geneva, Switzerland

## Abstract

**Background:**

Substance use among populations displaced by conflict is a neglected area of public health. Alcohol, khat, benzodiazepine, opiate, and other substance use have been documented among a range of displaced populations, with wide-reaching health and social impacts. Changing agendas in humanitarian response-including increased prominence of mental health and chronic illness-have so far failed to be translated into meaningful interventions for substance use.

**Methods:**

Studies were conducted from 2006 to 2008 in six different settings of protracted displacement, three in Africa (Kenya, Liberia, northern Uganda) and three in Asia (Iran, Pakistan, and Thailand). We used intervention-oriented qualitative Rapid Assessment and Response methods, adapted from two decades of experience among non-displaced populations. The main sources of data were individual and group interviews conducted with a culturally representative (non-probabilistic) sample of community members and service providers.

**Results:**

Widespread use of alcohol, particularly artisanally-produced alcohol, in Kenya, Liberia, Uganda, and Thailand, and opiates in Iran and Pakistan was believed by participants to be linked to a range of health, social and protection problems, including illness, injury (intentional and unintentional), gender-based violence, risky behaviour for HIV and other sexually transmitted infection and blood-borne virus transmission, as well as detrimental effects to household economy. Displacement experiences, including dispossession, livelihood restriction, hopelessness and uncertain future may make communities particularly vulnerable to substance use and its impact, and changing social norms and networks (including the surrounding population) may result in changed - and potentially more harmful-patterns of use. Limited access to services, including health services, and exclusion from relevant host population programmes, may exacerbate the harmful consequences.

**Conclusions:**

The six studies show the feasibility and value of conducting rapid assessments in displaced populations. One outcome of these studies is the development of a UNHCR/WHO field guide on rapid assessment of alcohol and other substance use among conflict-affected populations. More work is required on gathering population-based epidemiological data, and much more experience is required on delivering effective interventions. Presentation of these findings should contribute to increased awareness, improved response, and more vigorous debate around this important but neglected area.

## Background

Substance use among populations displaced by conflict is a neglected area of public health. Displacement contexts are beginning to be recognised as important risk environments for the development of substance-related harms, such as HIV infection [1-3]. Increasing attention to the humanitarian needs of internally displaced persons (IDPs), urban displaced populations, and situations of protracted displacement, coupled with a recognition of changing demographic and epidemiological contexts, has resulted in calls for more attention to chronic illness [4,5]. Globally, substance use is an important cause of ill-health and mortality-alcohol alone accounts for some 4% of mortality [6] and is linked with a number of mental health problems including depression [7]. Growing interest in the mental health of populations displaced by conflict in recent years has provided little insight into substance use: most of the work focuses on post-traumatic stress disorder and depression [8-20]. A number of effective interventions exist for problem substance use [21-24], but little attempt has been made to adapt these interventions to populations displaced by conflict. The information base on which to base these interventions remains sparse.

A range of substance use has been described in different settings: Khat chewing in conflict-affected Somalia [25], alcohol drinking among urban internally displaced populations in Colombia [26], inhalation and injection of heroin and opioids among Afghan refugees in Pakistan [27-30], and oral benzodiazepines among war-displaced in Bosnia-Herzegovina [31]. Increased [32] or excessive substance use has been reported from some [26,33] populations displaced by conflict; most studies are limited by lack of comparative data with populations who have not been displaced. Associated health problems in non-displaced populations have been well documented [7,34-36]. In addition, specific problems documented from conflict-affected populations include alcohol-related suicides [37,38]; gender-based violence [39,40]; injection drug use-related risks (transition to injection while refugee in exile [41], increased HIV and other blood-borne virus (BBV) transmission [27-29], and TB treatment failure [42]); and disruption to household economy [43], exacerbating already high levels of poverty [44].

Substance use problems can develop in the country of origin, in transit, in temporary refuge, or in resettlement [45,46]. A variety of risk factors for developing problem substance use in these settings have been reported, including male gender [33], exposure to war trauma [47-49], displacement [32], and co-existing mental health problems [50], although the relationship between post-traumatic stress disorder (PTSD) and substance use is complex and not well understood [33,47]. The social, cultural, political and economic factors underlying these risk factors are even less understood. These elements make up the 'risk environment' in which substance-related harm may be promoted or inhibited [51]. Examples include: geographical and regional differences [52]; macro-economic changes [53]; limited alternative livelihoods[43]; poor governance [25]; involvement of (former) combatants in the production and use of substances [25]. Religiosity [9,54] (for diverse reasons [55,56]) may be partially protective. For populations displaced by conflict, the relationship between the humanitarian response to displacement and promotion of or protection from problem substance use may also be important.

The literature on interventions among populations displaced by conflict, particularly harm reduction interventions [41,57], and is even thinner. While methodological and ethical considerations are paramount [58,59], evidence-based interventions can be adapted from stable settings. Yet there are remarkably few examples in the literature, even the so-called 'grey literature' of agency reports and non-peer reviewed publications, with some notable exceptions such as work in Afghanistan with injection drug users returning from neighbouring countries [57].

One approach for both improving information from conflict-displaced populations and building experience of developing interventions is to promote the conduct of rapid assessments. Rapid assessment methods have been commonly used in both the substance use field [60,61] and humanitarian settings for the last two decades [62,63]. These methods show promise as intervention-oriented assessment methods [64,65]. Although the term is used to encompass a number of heterogeneous approaches, for the purposes of these studies we based our approach on an existing series of Rapid Assessment and Response (RAR) guides developed for use in the substance use field among stable populations [66-71]. The main emphasis of these methods is an attempt to collect qualitative data using shorter versions of more lengthy and in-depth ethnographic methods[72]. Features include rapidity (weeks to months from initiation to final report), intervention focus, use of multiple data sources, multi-sectoral and community based approach, continued triangulation of data and use of an iterative approach to hypothesis formulation and testing evolving throughout the data collection and analysis period [60,73-76]. We applied these methods in six heterogeneous populations: the findings will be presented here, and implications for interventions discussed.

## Methods

### Study populations

Six rapid assessments were conducted from August 2006 to January 2008. The studies concerned a diverse range of populations-IDPs, refugees, surrounding communities, returning populations, both in and out of camps, in urban and rural settings, in Africa (Kenya, Liberia, and Uganda) and Asia (Iran, Pakistan, and Thailand). Sites were selected by the commissioning agency (UNHCR) based on results of HIV Behavioural Surveillance Studies, reports from UNHCR staff and partners of problem alcohol and other substance use among the populations concerned, requests for guidance on possible interventions by practitioners.

The study sites are summarised in Table 1.

**Table 1:** Rapid assessments of substance use among conflict-displaced populations 2006-8

Country	Site	Study population	Living environment	Displacement type	Date
**Africa**					

Kenya	Kakuma camp and surrounding community	Refugees (Sudan 80%, Somalia 13%, other) and surrounding population	Camp	Protracted civil conflicts	4-30/9/2006

Liberia	Monrovia, Tubmanberg, Voinjama	Returned refugees and IDPs	Urban	3 years post civil conflict	18/9 - 11/10/2006

Uganda	Northern Uganda (Kitgum, Gulu, Pader) - 6 camps	IDPs	Camp	Protracted civil conflict	5-31/7/2007

**Asia**					

Iran	Tehran	Refugees (Afghanistan)	Urban	Protracted international conflict	01/06/2007 - 31/01/2008

Pakistan	North West Frontier Province - 5 camps; Baluchistan-Quetta	Refugees (Afghanistan)	Camp and urban	Protracted international conflict	10/6 -9/7/2007

Thailand	Myanmar border-3 camps	Refugees (Myanmar)	Camp	Protracted civil conflict	6-25/8/2006

### Aims and objectives

All studies aimed to describe the current situation with respect to substance use and related harms among the study populations, and to identify a range of interventions that could be feasibly implemented to minimise harms related to substance use, particularly HIV transmission.

The studies aimed to inform harm and risk reduction related to alcohol and other substance use (including the reduction of HIV transmission risks) to individuals, families and communities. Objectives were to:

1. Identify psychoactive substances that are considered to be of public health importance by service providers, policy makers, and affected populations

2. Describe the social, economic, political and cultural context in which substance use occurs

3. Describe the community's and service providers' understanding of: patterns of use, populations and settings most affected by substance use; benefits and harms associated with their use; reasons why some people may be protected or vulnerable to harms associated with the use

4. Describe existing resources and interventions relevant to substance use and related harms (including general health, HIV, mental health and psychosocial support)

5. Identify important gaps in knowledge requiring further research before interventions can be implemented

6. Outline priority interventions that can be feasibly implemented at individual, community and policy levels

For the purpose of these assessments, psychoactive substances were considered to include any natural or synthetic chemical-licit or illicit-that acts on the brain to alter emotions, thoughts, perceptions, or behaviours. Tobacco products were excluded.

### Methods and procedures

The methods and procedures used in each site are summarised in Table 2. Details are available in the individual reports. The selection of methods varied by setting depending on security and other logistic constraints, as well as the quality of available data and the amount of assistance. Following a literature review of relevant published and unpublished materials, all studies conducted key informant and focus group interviews. Interviews were conducted either by the researcher aided by an interpreter, or by a trained and supervised team of field workers. Researchers maximised the information given the time and logistic constraints available, aiming for adequate information on the range of relevant cultural experiences in the assessment population. As in other qualitative research in the substance use field, the aim is for cultural and not demographic representativeness[77]. A range of men and women from different culture and language groups, of different ages participated. In deciding on the sample size, assessment teams followed the principle of 'pragmatic redundancy' where data collection was stopped when teams were satisfied that core cultural beliefs had been represented when now no new information was found (data saturation) [78].

**Table 2:** Summary of methods by study

Study	Methods (KI = key informant interview, FG = focus group interview)	Sample size	Sample characteristics	Sample selection	Duration of field work
**Africa**					

Kenya	Literature review Mapping Direct observation Semi-structured KI FG Group discussion	6 sites 20 KI 14 FG (n = 5-12) 3 group discussions (n = 20-34)	Gender: female and male Age: 17-57 Ethnicity: >9 groups Expertise: Substance users; service providers; sex workers; young people; teachers; people living with HIV/AIDS; post-voluntary counselling and testing groups; health workers; pre-formed community groups	Mix of purposive pre-selection by agency staff and snowball sampling	27 days

Liberia	Literature review Semi-structured KI FG	3 sites 15 KI 5 FG (n = 4-7)	Gender: female and male Age: 17-58 Ethnicity: various, except Voinjama Loma only Expertise: CSWs, service providers, children affiliated to fighting forces, shopkeepers, substance user	Pre-selection by agency staff	24 days

Uganda	Literature review Direct observation Semi-structured KI FG	6 sites 13 KI 6 FG (n = 5-11)	Gender: female and male Age: 21-54 Ethnicity: Acholi (residents), other Ugandans (service providers) Expertise: camp leaders, members of camp committees, service providers, mother-child groups, women brewers, other camp residents	Mix of purposive pre-selection by agency staff and snowball sampling	27 days

**Asia**					

Iran	Literature review Semi-structured KI FG	41 KI 7 FG (n = 7-10)	Gender: female and male Age: 16-55 Ethnicity:Hazara, Tajik, Pashtun, Sadat, Fars and Baluch Expertise: substance users, service providers, students, female heads of households, construction workers, teachers, service providers	Mix of purposive pre-selection by community leaders and snowball sampling	120 days

Pakistan	Literature review Secondary data analysis Direct observation Semi-structured KI FG	14 sites 53 KI 23 FG (n = 5-6)	Gender: female and male Age: 16-40+ Ethnicity: Pashtun, Turcoman, Tajik, Uzbek) Expertise: community leaders, service providers, young people, substance users, former substance users and their relatives	Purposive pre-selection by agency staff	30 days

Thailand	Literature review Semi-structured KI FG	3 sites 36 KI 14 FG (n = 4-11)	Gender: female and male Age: 17-55 yrs Ethnicity: Karen, Karenni Expertise: service providers, community leaders, camp officials, community members, pre-formed community groups, substance users	Mix of purposive pre-selection by agency staff and snowball sampling	20 days

In addition, three studies conducted direct observations of sites relevant to substance use observing people's behaviours, people and objects present, making detailed notes afterwards. Local agency staff assisted in the selection of sites. One study (Kenya) also asked key informants to help map relevant places such as sites of alcohol production, use and sale, services and other facilities on a hand-drawn plan of the camp as well as leading group discussions with preformed community groups. One study (Pakistan) collected and analysed secondary data (drop-in facility data).

Initial meetings were held with community leaders to explain the purpose and rationale of the assessment, promote community involvement and in particular the community's role in follow up actions. Preliminary results were fed back in community meetings and action plans developed either as part of the initial process or subsequently once the results had been finalised.

### Analysis

Data analysis began in the field during the period of data collection. The data were collated into broad themes by each researcher in a matrix. Findings were reviewed at the end of each day by the researcher and field workers to identify emerging themes for further exploration in focus groups and with members of the community. The researcher then conducted further thematic analysis, including refining and categorising of themes, identification of linkages between themes and subthemes, search for negative or deviant examples, triangulation with other data sources, and quotes to exemplify the arguments, once the data collection was complete.

### Protection of participants

The studies were conducted as operational research to inform decision making with respect to interventions, and complied with UNHCR standard procedures. Verbal informed consent was obtained from all participants by reading a consent form in a language understood to the participant outlining: the purpose of the assessment; the use of the results; the confidentiality of the interviews; and the voluntary nature of the interviewees' involvement. Interviewees understood that results would be anonymous and no identifying information would be recorded or reported in any way. All attempts were made to conduct interviews in a private location where the conversation could not be heard. Where translators were involved in data collection they were either persons known to UNHCR or UNHCR field staff who had signed an interpreter's undertaking, which includes the maintenance of confidentiality. No identifying information was recorded in the project documentation. The studies were conducted for the purposes of improving service provision, resulting in better interventions in substance use, both for the communities who participated and for other similar populations. Funds were allocated from the outset for project implementation in each of the study sites. Procedures to respond to adverse events (to protect both participants and researchers) were established prior to data collection, including referral for further care if requested. No adverse events were recorded.

## Results

Key qualitative findings are summarised here by country. Detailed findings can be found in the individual reports.

### AFRICA

#### Kenya

Kakuma Refugee camp is found in the arid north-western part of Kenya near Kakuma town. At the time of the assessment there were approximately 100,000 mainly Turkana people in Kakuma town, and close to 100,000 refugees in Kakuma Refugee Camp. The camp was established in 1992 to house Sudanese refugees; at the time of the assessment there were refugees from 9 countries-the Sudan (80%) and Somalia (13%), and smaller numbers from Ethiopia, Uganda, Rwanda, Burundi, the Democratic Republic of the Congo, Eritrea, and Namibia. A large programme of repatriation to Sudan was underway. Access to health, HIV and other services for the refugee population was satisfactory; there was also an alternative income generating programme available for women sex workers and alcohol brewers offering micro-credit initiatives for small businesses such as catering services, hairdressing, small foods and soft drink kiosks, peanut butter production, and tailoring.

Alcohol production and use was widespread. Fermented cereal-based *busaa *and the stronger distilled *changa'a *were both popular. In addition, khat (legal) and (clandestine, illegal) cannabis use was reported. Other substances included petrol or organic solvent inhalation. Injection drug use was not considered a significant public health problem: injecting of pharmaceuticals (mainly benzodiazepines) was thought to be uncommon, and heroin or cocaine thought to be rare if not completely absent in the camp and the local community.

Alcohol was seen as useful to "kill time" as well as being important for enjoyment and socialisation. Alcohol production and sale (whether or not associated with sex work by women) was an important source of income in the camp and in the local community. A number of problems were reported, however. The distilled product was illegal and producers subject to intermittent police raids. Violence, particularly gender-based violence, was perceived to be linked to alcohol use. Other perceived problems included mental health concerns, family disruption, and diversion of scarce household resources.

Alcohol use was linked to sexual behaviours that placed people at risk of HIV/sexually transmitted infection (STI) transmission and unplanned pregnancy, both within and between the refugee and surrounding populations. As one woman explains:

*"Drinking makes me feel sexually aroused. I may then sleep with anybody without caring about precautions" *(Woman brewer/sex worker during a group discussion in Kakuma Town).

Unsafe sexual practice was confirmed by this man

*"People who take drugs get reckless with sex because they don't care who they go to bed with. They don't even use any protection to protect them from infections. In addition, they have multiple partners and every day you will find a man with a different woman. The drug user sees the world as if it has no end and they feel so happy" *(Man from Equatoria, Sudan, current alcohol and khat user, former petrol and cannabis user).

Local community members felt that distilled alcohol brewing had increased because food rations (maize and sorghum) provided a good source of raw materials from which to produce the drinks, either by the refugees themselves or by the surrounding community: *"We buy the food rations from the Equatoria, Nuer, Dinka, Acholi from Uganda. The Ugandans produce the best chang'aa [distilled alcohol]. The communities that do not produce are the Congolese, Ethiopians and Somalis" *(Man during focus group with local Turkana community group leaders).

For one participant, alcohol production and use changed over time under the influence of different (external) groups, and now particularly under the influence of refugees: *"During the European time, many clubs existed where people sold and drank busaa....People later improved on the technology of brewing by distilling busaa to changa'a. The brewers are local people, mostly women who produce both busaa and changa'a. ... When the refugees came, they (particularly the Sudanese) brought their own technology and further improvised on the brewing of the local drinks." *(Man, senior local community member).

Limited alternative livelihoods, particularly for women, promoted production of alcohol: *"I brew because I want my children to survive. When my customers buy my brew and buy my body, even if I die, my children will inherit my brewing business." *(Woman brewer/sex worker during a group discussion in Kakuma Town).

(Sub)-cultural norms surfaced as important in promoting or inhibiting alcohol use. For example, for young people, use of alcohol was associated with their identity. *"To be a nigger, you've got to take alcohol and cigarettes" *explained one male student during a focus group. On the other hand, alcohol use among unmarried southern Sudanese men and women is not accepted, and thought to be exceedingly uncommon.

#### Liberia

2003 marked the end of 14 years of civil war that resulted in the death of approximately 250,000 people, accompanied by the near total destruction of infrastructure, and the beginning of the return of some 340,000 refugees and 500,000 IDPs. At the time of the assessment (2006) access to health, HIV and education services around the country were limited, fragmented, and supported largely by international non-governmental organisations (NGOs). The population experienced breakdown in water and sanitation systems, widespread food insecurity, unemployment and limited livelihood options. Seventy six percent of the population lived below the poverty line of US$1 per day, with 52% living on less than US$0.50 per day. Out of a total population of around 3.5 million, unemployment was almost one million people, over 80% of the labour force. Between a third and a half of the country's population lived in the capital Monrovia, where security was seen as better. Furthermore, economic opportunities were greater than in rural areas where there is little culture of growing cash crops outside the decimated plantation economy. In the capital city there was an active informal sector consisting mainly of small subsistence enterprise, for example food stalls, petty trading in dry goods, used clothing and domestically consumed agricultural products like beans, sugar cane, palm oil and vegetables.

Alcohol and cannabis were considered easily available, relatively cheap and widely consumed by men and women of all ages, with an important role in socialisation and relaxation. Distilled cane juice liquor was cheap (around US$0.5 to 0.20 for a shot glass) and consumed in bars or at street stalls. In addition locally produced palm wine is popular, available for around US$0.80 a litre bottle. Locally produced commercial spirits such as 'Godfather' whiskey, 'Bye Bye' tonic wine and 'Superman' dry gin were readily available. Beer was another higher status drink, as one respondent told us: "*beer is drunk like water, assuming that people can afford it*".

Cannabis was typically smoked in a rolled or cigarette for around US$0.10 (Liberian $5.00) for one 'wrap' or 'parcel', enough to get 2-3 people intoxicated. It was also cooked in soup and brewed as a tea as an intoxicant and as an appetite stimulant. Cannabis was often (and sometimes confusingly) referred to as 'opium'. It was seen as an important cash crop for some counties. In Voinjama, the use of herbal cannabis has become such a problem among young people that one high school had banned children from wearing dark glasses, used to mask the red eyes typical of cannabis intoxication. Ex-combatants and their friends are typically perceived as the main sellers and users of cannabis. One young person, however, claimed that cannabis use was common among many young people aged 12-25, not just ex-combatants. For him, all young people had been affected by the war, either through combat, loss of home and family or social dislocation, and had started cannabis use to be brave and strong to fight or just to meet their everyday difficulties. According to him "*now they take it to stop the bad dreams*."

The benzodiazepine, diazepam, known as 'ten-ten' 'five-five' and 'bubbles' was purchased without prescription from some pharmacies and reportedly used during the civil war by combatants and other young people affiliated to fighting forces to make them 'fearless' and 'brave'. It was relatively cheap at US$0.10 or less for one 5 mg tablet. Several sex workers interviewed reported that it is used in bars as a 'date rape' drug, with men slipping the substance into the drink of women without their knowledge or consent. Other men allegedly use it "*to be brave and for courage in order to commit robbery*."

Different forms of cocaine were also available, as well as heroin, although high prices may prevent more popular use of these substances. A cocaine and cannabis smoking mix called a 'dugee' appeared to be more common (perhaps because it is cheaper at around US$5.00) and was reported to be typically consumed by inhaling using the 'chasing the dragon' method. No respondents reported injecting drugs, although injection drug use was reported second hand in returned refugees.

Substance use was believed by many respondents to be problematic because it promoted health problems and violence, particularly gender-based violence. An urban fear of substances and crime-associated with ex-combatants-pervaded Monrovia. One respondent explained: "*Each area has its own ghetto where people who are of criminal nature, who take drugs, who do things unlawfully, they get together and stay in these areas."*

Endemic poverty and unemployment, ongoing insecurity, police corruption, gender and other structural inequalities were all considered to promote problem substance use. In addition, combat and displacement experiences may promote use "*to dull their fears and anxieties and to commit heinous atrocities*" explained one respondent. There were no specific substance use treatment services. Access to general health, HIV and education services-which may minimise problems resulting from substance use-was limited.

#### Uganda

At the time of the assessment (2006), 20 years of civil war in northern Uganda had displaced more than 2 million people into more than 100 IDP camps. Most of the displaced were still living in the 112 long standing over-crowded 'mother camps' in which access to health care and other services was limited. As part of the government's decongestion policy, some 350 smaller 'decongestion camps' or 'transit settlements' were established in 2005 as the first step towards return to ancestral lands; less than half of the displaced population had moved out due partly to lack of peace agreement and services in the new camps. Reluctance to move may be particularly pronounced among those requiring assistance (including alcohol dependent people) and younger people now unfamiliar with more traditional rural lifestyles.

Access to health care and other services in these camps was limited. Alcohol was readily available, its use widespread and considered an important public health and social problem. In addition, some cannabis use was reported, although its use was hidden due to threat of punishment and it was seen as a less important problem than alcohol from the community perspective.

As elsewhere, alcohol was used for recreation and pleasure. Respondents associated a number of problems with alcohol use, including unsafe sex, health problems (such as TB, lack of adherence to HIV treatment, mental health problems, and possibly suicide), dependence, and interpersonal and gender-based violence. Household financial problems, resulting from indebtedness and trading family rations and other goods for alcohol, left families short of food and children hungry.

In the context of limited livelihood options, alcohol brewing was considered an important source of income for many women. As one woman explained during a focus group with women brewers: "*we prefer to brew alcohol, it is our culture and easier than other work, we have no strength for other work, we can brew at home, and there is always a good demand." *Sometimes income generating was a collective activity. Another camp resident continues:" *I am ... part of a group of 7 women who all distil *arege *as a full-time job. We help each other in turn to brew. This is called *kalulu, *communal reciprocal labour. The name of our group is called *pii aye kwo, *meaning 'water of life'. I would like another form of work if possible, but there is nothing else available here"*.

Many respondents, both men and women, drew causal links between dispossession and alcohol use. Dispossession promoted alienation, idleness and loss of traditional gender roles among men. As a result, since alcohol was readily available and its use culturally accessible for men, alcohol use was increasing among men. *"Men have nothing to do, now many even choose not to work in the fields, they have too much time on their hands. Their other responsibilities have been eliminated by camp life and they have become idle." *explained one woman camp resident. As a result, cultural norms were changing, as one woman explained: *"now there are no rules for drinking alcohol"*. In turn, this promoted disrespect towards male clan elders and leaders. As one youth said, *"how can I respect these older men when I see them becoming drunk and falling down in the dirt." *The net effect of these adverse consequences may be a disruption to community cohesion, possibly inhibiting community recovery capacity.

### ASIA

#### Iran

For more than 20 years Iran has hosted refugees fleeing neighbouring Afghanistan-mainly Hazara, Tajik and Uzbek ethnic groups as well as some groups of Pashtun ethnicity, both Shiite and Sunni Muslim adherents. At the time of the assessment, there were close to one million registered Afghan refugees living in urban, semi-urban and rural areas of Iran, of whom only around 26,000 live in camps. There were an estimated further one million undocumented Afghans. Refugees are permitted access to basic education and health care on the same basis as Iranian citizens. Service utilisation by Afghans was thought to be low due to a combination of barriers such as poverty, lack of awareness, and perceived discrimination, as well as fear of being identified by authorities. Iran is an important transit route for opiate trafficking: an estimated 40% of Afghanistan's opium production passes through Iranian territory, some of which is absorbed locally [79].

Opiates were believed to be readily available and their use widespread among Afghan refugees, although illicit and not always socially and culturally acceptable. The main substance used was opium (inhaled using the 'chasing the dragon' method), consistent with pre-displacement patterns of use. Patterns of use were changing. Use among young people and women was increasing. Newer opiates were becoming more popular, such as heroin, Iranian "crack" and crystal (highly concentrated forms of heroin), and there was some transition to injection. Nevertheless, respondents perceived opiate as less prevalent among the Afghan refugee population than the host population. Alcohol use was believed to be relatively rare, partly due to religious proscription and greater cost than other substances. Cannabis use (in the form of hashish) was considered common particularly among young people. Additionally, there was some amphetamine use reported among young people.

A number of benefits to opiate use were reported: pain relief, pleasure and socialisation. Problems cited included criminal activity to support substance use habits, involvement in dealer gangs, fights and robberies. Behaviours risky for HIV, STI and BBV transmission were reported, including sharing of injecting equipment, unprotected sex, and exchange of sex by women for substances. At the household level, family disruption and divorce, gender-based violence (such as fights around diversion of household resources for substance purchase by males, early marriage of girls either for money or as escape from stressful environment), family poverty and malnutrition, and health and mental health problems of users and family members.

Whereas tight non-substance using social networks among Afghan refugees were considered partially protective against problem substance use, respondents believed that a number of factors might promote substance use and related problems. Examples included: feelings of loss, distress, pain and suffering; curiosity, boredom, influence of social networks, and expectations of enjoyment (particularly young people); ready availability of opiates; involvement in sales networks and limited alternative income; lack of other recreational activities. Young male garbage pickers (13-17 years of age) were seen as particularly vulnerable to substance use and related harms. As a result cultural norms were changing among the displaced community, influenced by local patterns of use among surrounding populations, social marginalisation and economic exclusion of Afghans. Although there are a number of health, HIV, and substance use treatment services in Iran, lack of awareness, stigma, misinformation, fear of being reported, perceived discrimination, cost, and concerns about confidentiality limited utilisation of these existing services by Afghans.

#### Pakistan

At the time of the assessment (2007), Pakistan was home to approximately 3 million Afghans, less than half of whom were living in UNHCR-supported long-term refugee camps (called 'refugee villages') along the border; the remaining were dispersed both in urban and rural settings, and not in receipt of support from UNHCR. A major repatriation exercise was underway, with the eventual aim of closure of the refugee settlements. As a result, health and other services were being scaled down. From 2001 nearly 3 million Afghans had returned as part of the UNHCR-supported facilitated voluntary return programme. At the time of the assessment numbers were dwindling due to continued insecurity and lack of shelter in Afghanistan. Unregistered Afghans were considered illegal and subject to involuntary deportation.

The main substance classes used were opiates (mainly opium), cannabis (hashish) and tranquilisers (benzodiazepines). Opium was used by men and women; it was mainly smoked or sometimes eaten or drunk in the form of tea. Hashish was seen as used by men whereas tranquilisers were used by women. Alcohol use was seen as uncommon and mostly home-brewed from sugar-cane or grapes and used by young people. Although each refugee 'village' context was distinct, substance use patterns were characterised as a continuation or exaggeration of pre-displacement use modified under the influence of patterns of availability and village livelihood options. The urban displaced were perceived to be particularly influenced by local patterns of use. For example, in urban but not rural areas substances were sometimes injected, reflecting the substance use patterns of the host population. Respondents believed however that the estimated prevalence of injecting among Afghan displaced was still low. A range of problems were believed to be linked with opium including dependence (although this was felt to be rare), financial impacts, incarceration and child neglect. Injection drug use was linked to HIV and other blood borne virus transmission as well as abscesses. Gender-based violence was associated with shortage of money for substances including hashish and opium: one third of the women interviewed said that they knew someone who had a serious problem with hashish and gave accounts of domestic violence associated with its use.

Respondents believed that limited skills, education and employment opportunities promoted substance use. Women balancing livelihood and childcare responsibilities described giving opium to children to keep them quiet; this culturally acceptable practice was considered traditional and widespread. Religious norms proscribing substance use, especially alcohol, were seen as potentially important in preventing greater problem substance use. Some substance users had access to specialist substance use services in urban areas, although utilisation rates were thought to be lower than the local population; no specialist services were available in the villages.

#### Thailand

Refugees fleeing more than 50 years of civil war in Myanmar have been living in Thailand since the early 1970s. There are approximately 150,000 refugees (both registered and unregistered) living in 9 camps along the Thai-Myanmar border, in addition to several million undocumented and documented migrant workers. A programme of third country resettlement, mainly to the USA, was underway. Access to primary health care and education was considered good; in addition there is abstinence based residential substance use treatment programme in the camps. Health indicators (mortality rates and malnutrition) are comparable to the host population, whereas on the other side of the border in eastern Myanmar these remain high.

Alcohol was the most important substance-related public health and social concern. It was cheap and readily available, particularly an illicitly produced and sold home-brewed distilled rice liquor. A number of other substances were mentioned including *ya ba *(tablet form of methamphetamine and caffeine), diazepam, cough syrup, and opiates (mainly a smoking form of opium), as well as cannabis. Inhalant use of glues by young people in Mae La and Ban Mai Na Soi was reported. Use of all these substances was considered less prominent than alcohol.

Most adult men were believed to drink alcohol: alcohol use was described as a culturally acceptable and appropriate response to the stressors of displacement for men. As elsewhere, enjoyment and socialising were seen as important benefits of alcohol use. In addition to negative health effects (which many participants thought were made worse by the addition of adulterants), dependence, high risk sexual behaviour (associated with in- and out-of camp mobility), family and neighbourhood disruption, and gender-based violence were perceived to be linked to alcohol use.

Restricted movement, education, and employment opportunities were seen to drive a sense of hopelessness and idleness among men. Coupled with ready availability and social acceptability of alcohol drinking, this was believed to result in high levels of alcohol use particularly among men. Cultural norms were thought to be changing with increased use among young people and women. One man explains: *"Young people have no hope, no work, no further study and no future. They have three choices, they can leave the camp and look for work, they can lead a traditional life which means they will have lots of babies, or they can drink alcohol." *As in Uganda, dispossession was an important element, as one resident of Ban Ma Nai Soi explained *"we have lost our traditions, our property, our belongings and our country. Here we have a restricted limited life so we drink."*

## Discussion

The relationship between substance use and harm is complex and context dependent [80].

A number of elements of the displacement context may be important in facilitating substance-related harm. For example limited access to health services may influence the development of harms related to the substance uses (for example untreated alcohol-related injuries); lack of condoms or needles and syringes may facilitate risky behaviours such as unsafe sex or injection. Consistent with the public health approach, the end point is minimisation of substance-use related harms. This does not ignore the perception in some communities that substance use may have important social functions. Indeed the relationship between social cohesion and substance use is not explored. The combined effect of substance use problems may inhibit community capacity to recover from conflict [81], yet some types of substance use may be important for social cohesion in some settings. On the other hand, tight social networks were considered protective against problem substance use in some settings (such as Iran). The relationships between substance use, social cohesion and community recovery capacity are areas for further study.

More work needs to be done on developing effective interventions, ones that address both proximal and more distal determinants of problem substance use. Nevertheless, a number of points for intervention can be identified, based on interventions that have been developed in non-displaced populations. The minimum interventions have already been described [24]. They should include screening and brief intervention for high risk alcohol use, for which there is good evidence of effectiveness in other settings [21]. Identification and treatment of severe mental illness (as both a cause and consequence of substance use) should also be instituted. In addition, targeted provision of condoms and needles and syringes may be indicated. Primary health services should be capable of managing withdrawal and other acute problems.

Expanded interventions can include behaviour change communication to reduce HIV risk especially in those most at risk (for example women brewers, sex workers, and their clients in Kakuma, Kenya). More comprehensive peer-outreach needle-syringe exchange programmes and hepatitis B vaccination programmes among injection drug users, which have been shown to be effective in other settings [23,82] may be considered among conflict-displaced populations. Well evaluated community mobilisation strategies may promote cultural relevance, acceptability and sustainability of interventions, and have been shown to be effective in some settings [21,83]. Despite their popularity among many service providers and community groups, general public information campaigns and school-based education for primary prevention programmes have been shown to be ineffective to reduce alcohol-related harm [21].

Finally, complex interventions include access to comprehensive treatment services for mental health problems as both a cause and consequence of substance use and for substance use. Examples include cognitive behavioural and drug therapy for alcohol withdrawal and relapse prevention [21], and opiate agonists for opiate dependence [23,84]. Mental health assessments should include information on substance use. As far as possible substance use, HIV and other blood borne virus prevention, treatment, care and support should be integrated into primary health and community based services.

There are a number of limitations to these rapid assessments that need to be taken into account when interpreting the findings. Firstly, qualitative approaches provide nuanced information about individuals and communities at the time that the study is conducted, but conclusions cannot be generalized to other conflict-displaced populations or to the same population at a different time. This is particularly important in a setting of high population mobility, as in the six studies presented here.

Secondly, qualitative methods will not provide population-based estimates of the proportion of the population affected by areas of interest, nor any epidemiological certainty about risk factors or substance-related harms. There was a marked lack of quantitative data available for secondary analysis in all the study sites (with the exception of Pakistan where one relevant health services data set was found providing some limited data for analysis). Population-based methods such as household surveys may be needed to obtain quantitative data on these key issues, but can be compromised by fluid populations and marked disincentive to disclosure due partly to stigma associated with substance use among affected populations [9]. More work is required on obtaining reliable population based estimates of substance use and epidemiology of risk factors and related problems in these populations, as well as linking individual STI, HIV and BBV risk to population prevalence.

Finally, rapid assessment methods do not allow for a fully iterative exploration of the topic and examination of new issues as they came up. Most of the studies were conducted with a field work period of around four weeks. A more in-depth exploration may have highlighted more issues or allowed a more detailed analysis and ranking of the issues. Time constraints meant that the samples were heavily influenced by pre-selection. In addition, many populations were large and diffuse: we would expect that the information from a closed camp community such as Kakuma may be more culturally representative than a study in two urban areas of Liberia. The use of external actors unknown to the community did not readily facilitate examination of very stigmatised or penalised activities for which there are marked disincentives for disclosure (such as injection drug use in many settings). The degree to which communities could be engaged in the process was curtailed, and participation was limited to pre- and post-assessment community meetings. Execution of the studies among war-affected populations means that logistic and security constraints are to be expected, and may have affected the quality of the data.

The studies were all intervention-oriented, and the limitations highlight the tension between producing practically relevant work and scientific rigour. This tension is perhaps more prominent in humanitarian/relief/studies of forced migration than in other fields [59]. Nevertheless, we believe that credible and programmatically relevant information was obtained. The studies provided an overview of the populations' understandings of patterns, contributing factors, and consequences of substance use, thus permitting programmatic recommendations to be made.

**Figure 1:**
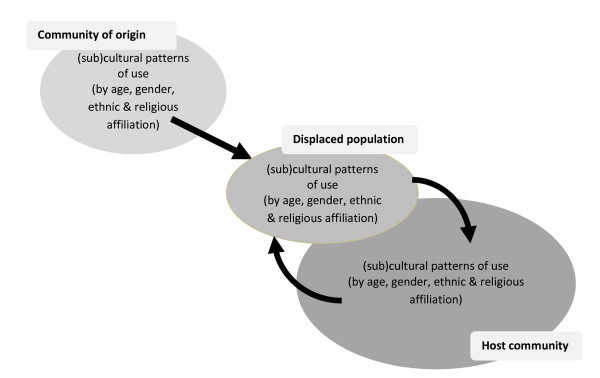
Changing patterns of substance use in conflict-displaced populations

Observations about the public health magnitude of substance use problems among the populations studied, or whether substance use and related problems is greater among these displaced populations than their community of origin or the host community, cannot be made. These studies do suggest however that substance use in conflict-displaced populations can be a continuation or exaggeration of pre-displacement patterns, or similar to the host population, or a mixed picture (Figure 1). For example, the suggestion from Iran is that patterns of opiate use among Afghan refugees are intermediate between origin and host patterns of use. As in other (non-displaced) populations, we would expect that patterns of substance use will vary also by sub-group, such as age, gender, ethnic and religious affiliation.

Factors that mediate these observed transitions-why, when, and under what conditions will populations and subgroups change patterns of substance use-are not clearly understood. Proximal facilitators may include ready availability of alcohol and other substances, and psychological triggers such as alleviation of emotional reactions associated with loss and adjustment. Changing social networks and cultural controls of substance use may also promote change. In addition, the studies suggest that a number of underlying elements of the displacement context may be important, such as restriction in movement, limited livelihoods, dispossession, and a sense of hopelessness. In particular, the findings suggest that substance use problems exploit the underlying power fault-lines in the community-be they along gender, ethnic, or economic lines. For example, many of the studies reported here link gender-based violence to alcohol intoxication (usually by men directed towards women). Cultural expectations and patterns of behaviour of men who drink to intoxication play an important role here, along with situational and individual factors [85,86]. In Pakistan where drinking alcohol is not so common, gender-based violence is also linked to substance use, but in this case it is reported as related to seeking money for substance purchase rather than intoxication with alcohol, highlighting the importance of the socio-cultural context in which violence occurs.

Although there is good evidence that regulation of access to alcohol (age for purchase, density of drinking establishments) and increased taxation minimises harms associated with commercial alcohol use, enacting and developing effective policy is a much more complex endeavour where there is a large illicit market, and where restrictions on commercial alcohol availability may promote illicit trade [21]. The illicit status of the substance was not identified as an important factor in protecting substance use problems by respondents. Nonetheless, it is suggested to address the availability and use of methanol where this is noted to be problematic, (for which there is evidence of effectiveness elsewhere) [21].

The observations of use of food rations other than for sustenance is not new [87], but it is the first time that it has been documented in the public domain as facilitating alcohol production and use in the community. However, selling or trading of rations is a recognised coping strategy among displaced populations [88]. The effectiveness of alternative livelihoods programmes implemented in some settings (e.g. northern Uganda and Kakuma, Kenya) on substance use problems has not been studied.

The populations included in these assessments have been displaced over long periods of time. Conclusions from these studies will therefore inform post-acute phase interventions (in which basic needs have been met). The final selection, design, monitoring and evaluation of the intervention will be context dependent, and demands community engagement [89]. A phased approach should be taken [90] to implementation interventions (Table 3): choice of interventions should be context specific, guided by baseline assessment. Interventions will need to incorporate systematic human resources capacity building efforts with an emphasis on practical skills-based training programmes. Changing population dynamics, such as movements in and out of camps, are the norm in most humanitarian settings [91], and must be taken into account. Although it is indeed difficult to make recommendations on future planning given uncertainty about the future (camp closure, repatriation), incorporation of substance use interventions into return, resettlement, and repatriation planning is an important response. As the information base for substance use interventions in these populations is thin, any intervention should be well monitored and evaluated, and the experience disseminated. Interventions will need to be implemented within a supportive and strategic policy framework-advocacy for partners and funds may need to accompany policy development and strategic planning processes. Efforts may need to include advocacy for inclusion of displaced populations in national policies and plans.

**Table 3:** Core interventions to minimize substance-related harms in populations displaced by conflict (adapted from [81,90])

Phase	Intervention
Minimum	Screening and brief intervention for high risk alcohol use Referral of severe mental illness Targeted provision of condoms and needles and syringes Management of withdrawal and other acute problems Continuation of opioid substitution therapy for those who commenced pre-displacement

Expanded	Targeted behaviour change communication for HIV/STI/blood-borne virus prevention Expanded needle and syringe programmes through outreach and hepatitis B vaccination programmes for injection drug users Thiamine provision for heavy alcohol drinkers Community mobilisation programmes to promote uptake of interventions and to decrease stigma

Complex	Substance use treatment services (including cognitive-behavioural and opioid substitution therapy) Incorporation of substance use treatment into: comprehensive mental health services (particularly depression); integrated chronic disease management (particularly hypertension/cardiovascular disease and TB); and HIV/STI programmes Incorporation of substance use prevention and management into gender-based violence prevention programmes

## Conclusions

These studies attempt to address a neglected area of public health importance among populations displaced by conflict. The conduct of these assessments has enabled the publication of a UNHCR/WHO field guide on rapid assessment of alcohol and other substance use among conflict-affected populations [92]. More work is required on gathering population-based epidemiological data. Nevertheless, they demonstrate that greater attention needs to be given to prevention and treatment of the harmful consequences of substance use in conflict-displaced populations. Substance use should not be seen as an isolated stand-alone issue: substance use interventions need to be considered as essential components of general health services (including TB control and chronic disease management), mental health and psychosocial support, HIV and STI interventions, and gender-based violence prevention.

With the exception of refugee camps in Thailand and some refugee villages in Pakistan, none of the settings had mechanisms in place to prevent or manage substance use problems. In some instances refugees living in urban areas used existing services in the host communities, but few were adapted to the needs of these displaced populations. In most of the sites there were generally weak regulatory mechanisms in respect to substance use reflecting the level of progress in the host country in addressing substance use. Furthermore, in humanitarian relief settings little attention is paid to substance use when other health and social problems are seen as more pressing.

Addressing substance use requires a concerted effort involving multiple sectors and several levels of engagement; it is not often seen by either humanitarian workers or donors as an integral component of the relief response even in post-acute response. This is compounded by the lack of adequate and comprehensive information on the harmful consequences including the health consequences of substance use in these settings, as well as lack of training of humanitarian workers in dealing with substance use problems. Humanitarian efforts should include advocacy for national health data collection efforts and prevention strategies to include displaced populations.

More experience is required collectively on how best to respond to substance use among conflict-displaced populations. Interventions need to be conducted and results disseminated. A global forum for exchange of experience, ideas, information and evidence is required. By presenting findings from these six assessments conducted among diverse populations, we hope to stimulate response among humanitarian actors.

## Competing interests

The authors declare that they have no competing interests.

## Authors' contributions

All authors contributed to the writing of the manuscript and read and approved the final manuscript. MA, EO, DM and AS lead the field studies, collected and analysed data; AB participated in the design and conduct of the study, field data collection and analysis in one of the sites; MS participated in the design and conception of the study, and coordinated its implementation; MvO participated in the design and conception of the study. NE participated in the design, conception, and analysis phases and prepared the final manuscript.
